# Toxicity of Carbon, Silicon, and Metal-Based Nanoparticles to the Hemocytes of Three Marine Bivalves

**DOI:** 10.3390/ani10050827

**Published:** 2020-05-10

**Authors:** Konstantin Pikula, Vladimir Chaika, Alexander Zakharenko, Anastasia Savelyeva, Irina Kirsanova, Anna Anisimova, Kirill Golokhvast

**Affiliations:** 1 Education and Scientific Center of Nanotechnology, School of Engineering, Far Eastern Federal University, Sukhanova 8, 690950 Vladivostok, Russia; chayka.vv@dvfu.ru (V.C.); zakharenko.am@dvfu.ru (A.Z.); golokhvast.ks@dvfu.ru (K.G.); 2 N.I. Vavilov All-Russian Institute of Plant Genetic Resources, B.Morskaya 42-44, 190000 Saint-Petersburg, Russia; 3 School of Natural Sciences, Far Eastern Federal University, Sukhanova 8, 690950 Vladivostok, Russia; saveleva.as@students.dvfu.ru (A.S.); kirsanova.ia@dvfu.ru (I.K.); anisimova.aale@dvfu.ru (A.A.); 4 Pacific Geographical Institute, Far Eastern Branch of the Russian Academy of Sciences, Radio 7, 690041 Vladivostok, Russia

**Keywords:** carbon nanotubes, ecotoxicology, flow cytometry, hemocytes, metal nanoparticles, bivalve mollusc, mussel, nanofibers, nanotoxicology, silicon nanotubes

## Abstract

**Simple Summary:**

The growing nanotechnology industry disposes of a variety of nanoparticles with different physiochemical properties in everyday life. However, the dependence of the safety and toxicity of nanoparticles on their physicochemical properties remains unclear. Bivalve molluscs represent an efficient model for the investigation of nanoparticle toxicity owing to their filtrating ability and feeding on particles suspended in the water. Moreover, the blood cells of bivalve molluscs, the hemocytes, have been suggested as a good analog test-object to mammalian immune cells, phagocytes. In this study, we used hemocytes of three marine bivalve species, namely, *Crenomytilus grayanus*, *Modiolus modiolus*, and *Arca boucardi*, to evaluate and compare the toxic effects of 10 different types of nanoparticles. We gave short-term exposure of the nanoparticles to the hemocytes and registered viability and changes in their cell membrane polarization by employing flow cytometry. Metal-based nanoparticles were the most toxic to the cells of all three tested bivalve mollusc species. However, the sensitivity to different nanoparticle types varied between species. Moreover, the registered cell membrane depolarization indicated an early toxic response and raised concern that chronic long-term exposure of nanoparticles (even if they were previously declared as safe) is a serious threat for aquatic organisms.

**Abstract:**

Nanoparticles (NPs) have broad applications in medicine, cosmetics, optics, catalysis, environmental purification, and other areas nowadays. With increasing annual production of NPs, the risks of their harmful influence on the environment and human health are also increasing. Currently, our knowledge about the mechanisms of the interaction between NPs and living organisms is limited. The marine species and their habitat environment are under continuous stress owing to the anthropogenic activities, which result in the release of NPs in the aquatic environment. We used a bioassay model with hemocytes of three bivalve mollusc species, namely, *Crenomytilus grayanus*, *Modiolus modiolus*, and *Arca boucardi*, to evaluate the toxicity of 10 different types of NPs. Specifically, we compared the cytotoxic effects and cell-membrane polarization changes in the hemocytes exposed to carbon nanotubes, carbon nanofibers, silicon nanotubes, cadmium and zinc sulfides, Au-NPs, and TiO_2_ NPs. Viability and the changes in hemocyte membrane polarization were measured by the flow cytometry method. The highest aquatic toxicity was registered for metal-based NPs, which caused cytotoxicity to the hemocytes of all the studied bivalve species. Our results also highlighted different sensitivities of the used tested mollusc species to specific NPs.

## 1. Introduction

The growing industry of nanotechnology generates a reasonable concern regarding the issue of safety and risk assessment for nanoparticles (NPs) [1,2,3]. This problem had created a new subcategory of toxicology, nanotoxicology [4], which is aimed to understand and explore the mechanisms of interaction between NPs and living organisms [5,6]. Despite the remarkable progress in this area, including multiple European Union (EU) and U.S. nano-specific databases and research projects, development of modern omics, and computational approaches [7], the potential influence of NPs to the aquatic environment is not fully understood and requires further investigation [8].

Cosmetics, electronic devices, pharmaceutical products, food supplements, clothing, coating, additives to some products like rubber and concrete, and other industrial and consumer products are the most common sources of engineered NPs reported in the environment [5]. NPs can enter the environment during their manufacturing, utilization, and disposal [9,10,11]. The potential release of NPs into the water has been estimated at up to 7% of the total global production [12].

Unique physicochemical properties of NPs provide a variety of their applications and, at the same time, make the assessment of their safety a very complex task [13]. The toxic properties of NPs mostly depend on particle size, surface area, surface chemistry, crystalline structure, method of synthesis, purity, or all together [8,14]. Moreover, the fate and behavior of NPs released in water depend on their physical, chemical, or biological transformations in the exposed environment [8,15,16]. Therefore, the evaluation of the aquatic toxicity of NPs requires a complex and multi-parametric study.

Traditionally, the aquatic toxicity of NPs has been tested in bioassays with different model organisms, such as bacteria [17,18,19], microalgae [20,21,22], crustaceans [23,24,25], sea urchin [26,27,28], bivalve molluscs [29,30], fish cell lines [31,32,33], fish [23,34,35], and amphibians [36,37]. The diversity of testing models allows us to compare the differences and similarities of toxic effects between species, which gives an opportunity to understand the potential hazards of NPs and the relationship between their toxicity mechanisms and physicochemical properties [38].

In this study, we selected bivalve molluscs as typical nearshore animals having their economic importance and ecological relevance among macrobenthic animals [39,40]. Bivalves are often used as sentinel species or environmental indicators owing to their ability to accumulate chemical contaminants, lack of mobility, and wide distribution throughout the coastal waters of the world [41,42]. Moreover, bivalve molluscs have been described as a good target group for nanotoxicology because of their ability to feed on suspensions as well as their highly developed ability for the cellular internalization of nano and microparticles [29,43]. The blood cells of bivalve molluscs, the hemocytes, play a key role in the innate immune response of a mussel and provide protection from foreign microorganisms and toxic substances [44]. As noted by Canesi (2012), the short-term in vitro effects of NP suspensions on mussel hemocytes, as well their mode of action, resemble those observed in mammalian phagocytes. This observation supports the hypothesis that, in bivalves, like in mammalian cells, cell-mediated immunity represents a significant target for NPs [29].

We have used a bioassay model comprising the hemocytes of three bivalve mollusc species, namely, *Crenomytilus grayanus* (Dunker, 1853), *Modiolus modiolus* (Linnaeus, 1758), and *Arca boucardi* (Jousseaume, 1894). The chosen species represent abundant bivalves of the Peter the Great Bay (Sea of Japan, Russia) [45]. *C. grayanus*, also known as Gray’s mussel, lives 95–150 years attached to stable solid substrates [46]. *C. grayanus* is tolerant to a relatively low concentration of dissolved oxygen (3–9 mL/L) and can survive in freshwater up to 44 h [46]. *M. modiolus,* also known as the horse mussel, lives 20–45 years on rocks, boulders, gravel, pebbles, and sand, sometimes being submerged 2–3 body lengths into the sediment [47,48]. *C. grayanus* and *M. modiolus* are closely related bivalve species of the family Mytilidae, which are commonly used for heavy-metal monitoring in coastal waters of the northwestern Pacific [49,50]. It was reported that, despite a higher level of heavy metal accumulation, *M. modiolus* has a defense strategy that includes gradual detoxification and excretion of the pollutants from the organs [50]. The defense system of *C. grayanus* was reported as ineffective under chronic pollution because of the high load of heavy metals in the digestive gland and kidney [50]. *A. boucardi* is a bivalve of the family Arcidae, which lives on sand or rock–boulder bottom, often forming a joint biocenosis with *C. grayanus* [51].

In the bioassay with mollusc hemocytes, we used 10 types of common NPs, that is, two types of multiwalled carbon nanotubes (CNT-1, CNT-2), two types of carbon nanofibers (CNF-1, CNF-2), two types of silicon nanotubes (SNT-1, SNT-2), nanocrystals of cadmium and zinc sulfides (CdS, ZnS), gold NPs (Au-NPs), and titanium dioxide NPs (TiO_2_). The toxic influence of the NPs on the hemocytes was measured by the flow cytometry method. In recent years, flow cytometry, a well-known routine tool used for vertebrate research, has been applied to bivalve immunology to study hemocyte morphology and immune-related activities [52,53].

Some toxic effects of different types of NPs on marine bivalves were previously described [30,54,55,56]. Despite published data, much uncertainty still exists about the relation between physicochemical properties of NPs and their toxic effects in the aquatic environment. Our study aimed to evaluate and compare the difference in cytotoxicity and cell-membrane polarization changes of the hemocytes of three bivalves exposed to 10 types of NPs.

## 2. Materials and Methods

### 2.1. Nanoparticles

Carbon nanotubes and nanofibers were synthesized and characterized in the Boreskov Institute of Catalysis (Novosibirsk, Russia) [57]. The structural features of carbon NPs ware assessed by Raman spectroscopy in our earlier report [58]. The length of the carbon nanotubes was hundreds of times larger than the diameter, and the particles could cohere into the spheres up to tens of micrometers in diameter in water suspension.

Silicon nanotubes were kindly provided by the Department of Chemistry, Inha University Republic of Korea [59]. The samples had a significantly lower ratio of length to diameter compared with carbon nanotubes.

CdS and ZnS NPs were synthesized and characterized in the University of Mining and Geology, St. Ivan Rilski (Sofia, Bulgaria) [60].

Au-NPs were synthesized by Turkevich method with reduction by citrate at 100 °C [61].

TiO_2_ nanopowder was purchased from Thermo Fisher Scientific (Thermo Fisher GmbH, Kandel, Germany, CAS number 1317-70-0, product number 39953). Characteristics of all the NPs used in this study are represented in Table 1.

### 2.2. Hemocytes Preparation and Exposure

The molluscs *C. grayanus*, *M. modiolus*, and *A. boucardi* were collected in October 2019 from Novik bay (Peter the Great Bay, Sea of Japan, Russia). Mussels were maintained for three days in an aerated 50 L tank filled with natural seawater. In this study, we used 16 adult specimens of each bivalve species.

The hemocytes of the molluscs were prepared using the previously described protocol [62]. Hemolymph was withdrawn from the posterior adductor of the molluscs using 0.3 mL syringes containing 0.3 M sodium ethylenediaminetetraacetic acid (EDTA) salt solution for the prevention of hemocyte agglutination. The obtained hemolymph was centrifuged, and the supernatant was removed. The pellet was washed with 1 mL of Calcium and Magnesium Free Artificial Sea Water Solution (CMFSS) and centrifuged again. The obtained cell pellet was resuspended in sterile seawater (20 ± 2 °C; pH, 8.0 ± 0.2; salinity, 33‰ ± 1‰). The volume of obtained hemocyte suspension from one mollusc was approximately 2 mL. The counting of collected cells was carried out with flow cytometer CytoFLEX (Beckman Coulter, Indianapolis, IN, USA) using the software package CytExpert v.2.0. The aliquots of the hemocytes at the concentration of 8∙10^4^ cell/mL were prepared to the bioassay.

The working suspensions of the NPs were prepared for bioassays by the addition of the NPs to sterile seawater to obtain a concentration of 1000 mg/L. Before each series of bioassays, the working suspensions of NPs were sonicated with ultrasound homogenizer Bandelin Sonopuls GM 3100 (Bandelin Electronic GmbH & Co. KG, Berlin, Germany) using maximal intensity for 30 min.

The prepared cell aliquots were replaced in 96-well plates (50 µL per well) and kept in a laboratory fridge (4 °C) for 30 min. Then, the prepared and sonicated working suspensions of the NPs (1000 mg/L) were diluted with artificial seawater, and 150 µL of these suspensions was added to the cells to obtain the final concentrations of 1, 10, 100, 250, 500, and 1000 mg/L. Only artificial seawater was added to the group of control cells. The cells were incubated with the NPs in the fridge (4 °C). The measurements were performed after 2, 4, and 6 h of the exposure. At each time point, three biological replicates of the hemocytes from each species exposed to all used NP types at each applied concentration and the control group were measured by flow cytometry.

### 2.3. Flow Cytometry

The counting of alive cells and registration of changes in cell-membrane polarization of hemocytes were carried out with flow cytometer CytoFLEX using the software package CytExpert v.2.0 (Beckman Coulter, Indianapolis, IN, USA). The toxic effects were evaluated using specific fluorescent dyes (Molecular Probes, Eugene, OR, USA). A blue laser (488 nm) of the CytoFLEX flow cytometer was chosen as a source of excitation light. The excitation laser and emission filters were selected according to the recommendations provided by the manufacturer of the dyes.

The viability of the cells was determined by staining with propidium iodide (PI) according to the standard bioassay protocol [63]. The mechanism of PI action is the incorporation between DNA or RNA base pairs, whereupon the dye increases its fluorescence intensity by 20–30 times [64]. As PI is unable to penetrate intact membranes of living cells, the cells with dramatically increased fluorescent intensity in the emission filter FL1 (610 nm) compared with control can be regarded as dead cells and excluded from the counting [65].

Membrane polarization of the hemocytes was assessed by a lipophilic, positively charged fluorescent dye 3,3′-dihexyloxacarbocyanine iodide (DiOC_6_), which is capable of binding to membranes (mitochondria and endoplasmic reticulum) and other hydrophobic negatively charged cell structures [66]. In the case where the inner membranes of the cell became more electronegative compared with medium (hyperpolarization, that is, an increase in the membrane potential), the dye will be absorbed. If the membrane potential decreases and the cell becomes less electronegative compared with medium (depolarization), the dye will be removed from the cell [67]. The changes of hemocyte membrane polarization were registered as the mean fluorescent intensity of DiOC_6_ attached to alive cells in the emission filter FL2 (525 nm).

The measurements were performed at a flow rate of 100 µL/min for 30 s. The signals of forward and side light scattering were registered in FSC and SSC emission channels, respectively, to develop the two-parameter histograms of cell distribution by size and granularity. Before the measurement, each sample of the hemocytes was stained simultaneously with PI (15 μM) and DiOC_6_ (0.5 μM) and incubated in the dark on ice for 20 min. The stained fixed cells were used as a negative control.

### 2.4. Microscopy

Morphological changes of hemocyte cells were observed and captured by an optical microscope Axio Observer A1 (Carl Zeiss, Oberkochen, Germany).

### 2.5. Statistical Analysis

Statistical analyses were performed using the software package GraphPad Prism 7.04 (GraphPad Software, San Diego, CA, USA). The one-way repeated measures analysis of variance (ANOVA) test was used for analysis. A value of *p* ≤ 0.05 was considered statistically significant.

## 3. Results

### 3.1. Cytotoxicity

**Figure 1:**
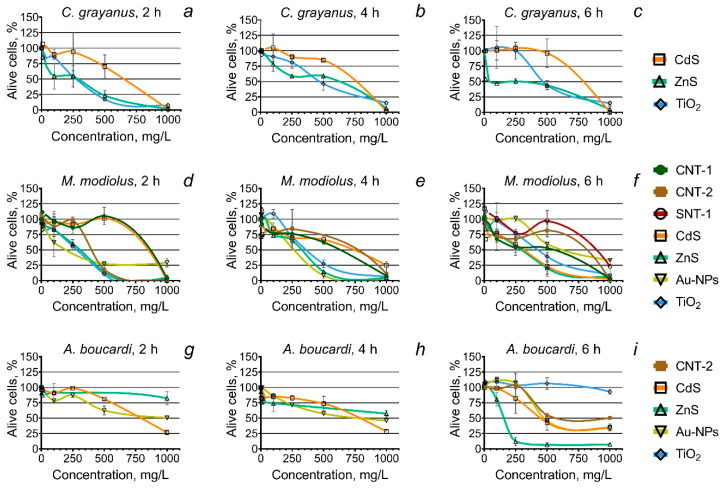
The viability of hemocyte cells of the molluscs *C. grayanus*, *M. modiolus*, and *A. boucardi* after the treatment with the nanoparticles (NPs); (**a**–**c**) the changes in the total number of alive *C. grayanus* hemocytes after 2, 4, and 6 h exposure to the NPs, respectively; (**d**–**f**) the changes in the total number of alive *M. modiolus* hemocytes after 2, 4, and 6 h exposure to the NPs, respectively; and (**g**–**i**) the changes in the total number of alive *A. boucardi* hemocytes after 2, 4, and 6 h exposure to the NPs, respectively. CNT, carbon nanotube; SNT, silicon nanotube.

The calculated concentrations of the NPs that caused 50% inhibition (EC_50_) of hemocyte viability are summarized in Appendix A (Table A1). The dynamic changes in the viability of hemocyte cells of three marine bivalve molluscs after 2, 4, and 6 h of exposure to the NPs are represented in Figure 1. For each tested species, only the NPs, which caused the observed changes in hemocyte viability, are represented in the figure.

Metal-based NPs (CdS, ZnS, Au-NPs, and TiO_2_) showed the highest cytotoxicity among the all tested samples. The CdS and ZnS NPs demonstrated a pronounced cytotoxic effect on the hemocytes of all three mollusc species (Figure 1a–i). Moreover, the toxicity of the ZnS NP sample for *C. grayanus* and *A. boucardi* increased significantly after 6 h of exposure (Figure 1c,i). The Au-NPs strongly affected the hemocytes of two species, namely *M. modiolus* and *A. boucardi* (Figure 1d–i), but did not affect *A. boucardi* (Figure 1a–c). The TiO_2_ NPs caused substantial mortality of *C. grayanus* and *M. modiolus* hemocytes (Figure 1a–f). For *A. boucardi*, however, the TiO_2_ NPs exhibited a relatively low cytotoxic effect, which was observed only after 6 h of exposition to the highest NPs concentration, 1000 mg/L (Figure 1i).

Overall, the hemocytes of *M. modiolus* were the most sensitive to all types of the NPs. Carbon nanotubes CNT-1 and CNT-2 demonstrated a cytotoxic effect on *M. modiolus* in all three measurements (Figure 1d–f). Besides, the sample CNT-2 significantly affected *A. boucardi* hemocytes after 6 h of exposure (Figure 1i). Moreover, only *M. modiolus* showed the toxicity of SNT-1 after 6 h of exposure (Figure 1h).

### 3.2. Membrane Polarization Changes

**Figure 2:**
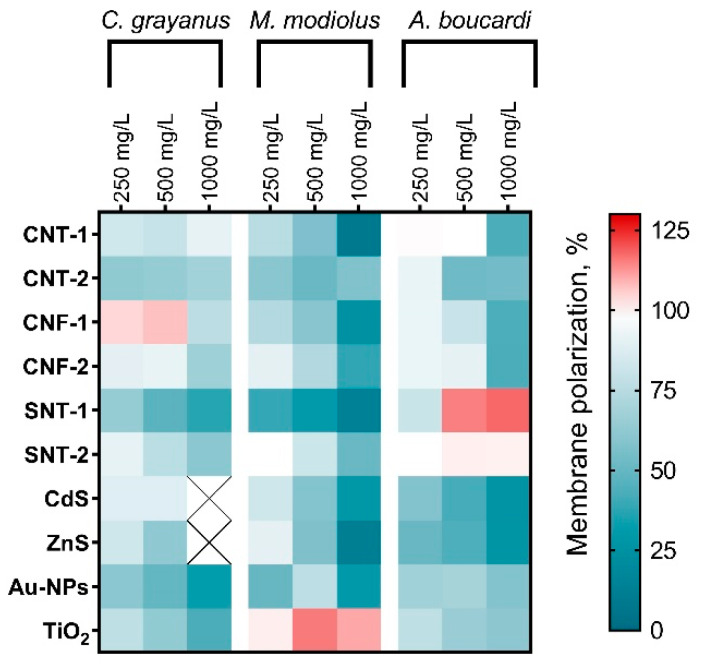
The changes of membrane polarization of *C. grayanus*, *M. modiolus*, and *A. boucardi* hemocytes after 6 h of the treatment with the NPs at concentrations of 250, 500, and 1000 mg/L. CNT, carbon nanotube; CNF, carbon nanofibers; SNT, silicon nanotube; NP, nanoparticle.

The influence of the NPs on membrane polarization of *C. grayanus*, *M. modiolus*, and *A. boucardi* hemocytes is represented as a heatmap (Figure 2). In Figure 2, we have shown the membrane polarization changes of hemocytes after 6 h of exposure with the highest concentrations of the NPs (250, 500, and 1000 mg/L). The mean fluorescence intensity of DiOC_6_ attached to the cells of the control group was taken as 100%.

For most of the tested NPs, we can notice the dose-dependent membrane depolarization. The sensitivity and reaction differed between the species and even hyperpolarization could be noted in some cases. The highest membrane depolarization under the influence of all NP samples (except TiO_2_) was demonstrated by the hemocytes of *M. modiolus* at the concentration of 1000 mg/L.

All carbon-based NPs (CNT-1, CNT-2, CNF-1, and CNF-2) had the lowest influence on *A. boucardi* hemocyte membrane polarization at the concentration of 250 mg/L, but significant depolarization was recorded at 1000 mg/L.

Silicon nanotubes SNT-1 caused the highest membrane depolarization of *C. grayanus* and *M. modiolus* hemocytes. At the same time, *A. boucardi* responded with hyperpolarization of hemocyte membranes under the influence of the sample SNT-1 and demonstrated no response to the influence of the sample SNT-2.

Among the metal-based NPs, the most pronounced membrane depolarization was caused by the influence of CdS and ZnS NPs. Depolarization of *C. grayanus* hemocyte membranes was not registered for these samples at 1000 mg/L as almost all the cells were dead (Figure 1a). Au-NPs decreased membrane polarization of *C. grayanus* and *M. modiolus* hemocytes higher than the polarization of *A. boucardi* cells. TiO_2_ caused membrane hyperpolarization of *M. modiolus* cells (Figure 2).

### 3.3. Visual Observation

**Figure A1:**
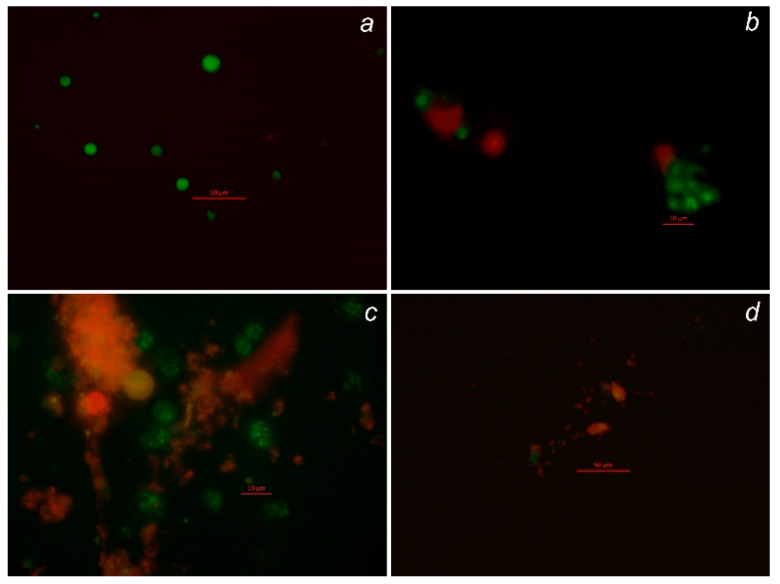
The hemocyte cells of the mollusc *C. grayanus* after 6 h of exposure with the NPs at the concentration of 1000 mg/L: (**a**) control; (**b**) CdS; (**c**) ZnS; and (**d**) TiO_2_.

**Figure A2:**
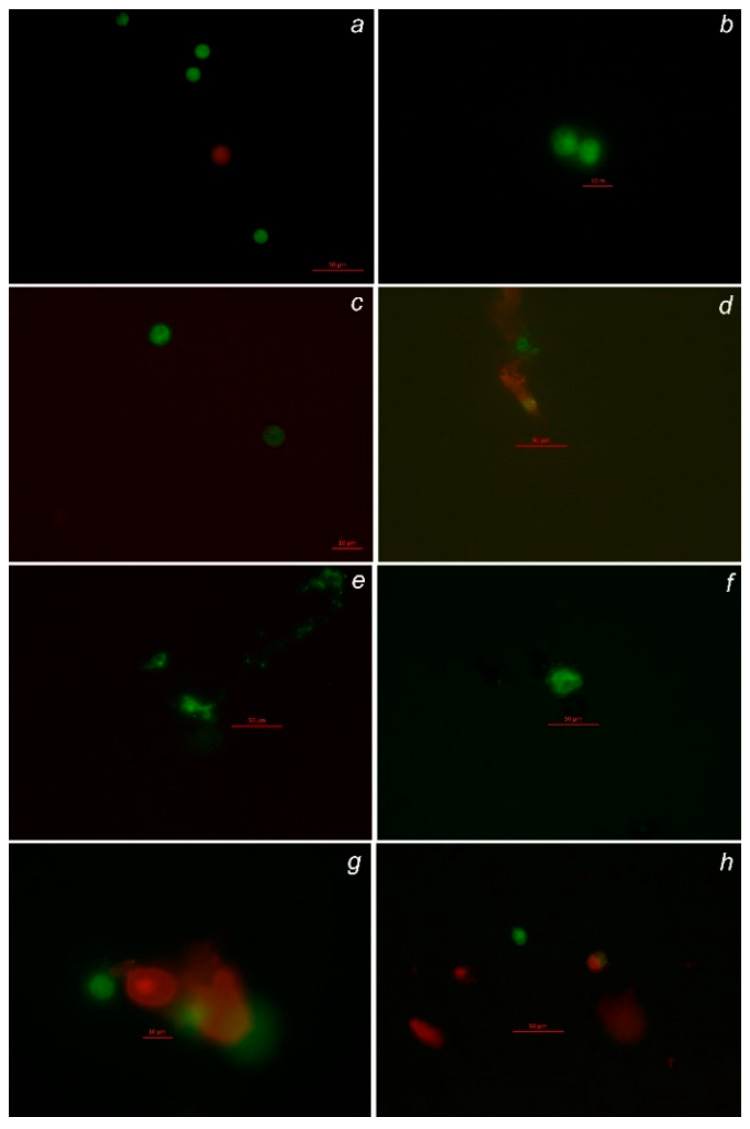
The hemocyte cells of the mollusc *M.modiolus* after 6 h of exposure with the NPs at the concentration of 1000 mg/L: (**a**) control; (**b**) CNT-1; (**c**) CNT-2; (**d**) SNT-1; (**e**) CdS; (**f**) ZnS; (**g**) Au-NPs; and (**h**) TiO_2_.

**Figure A3:**
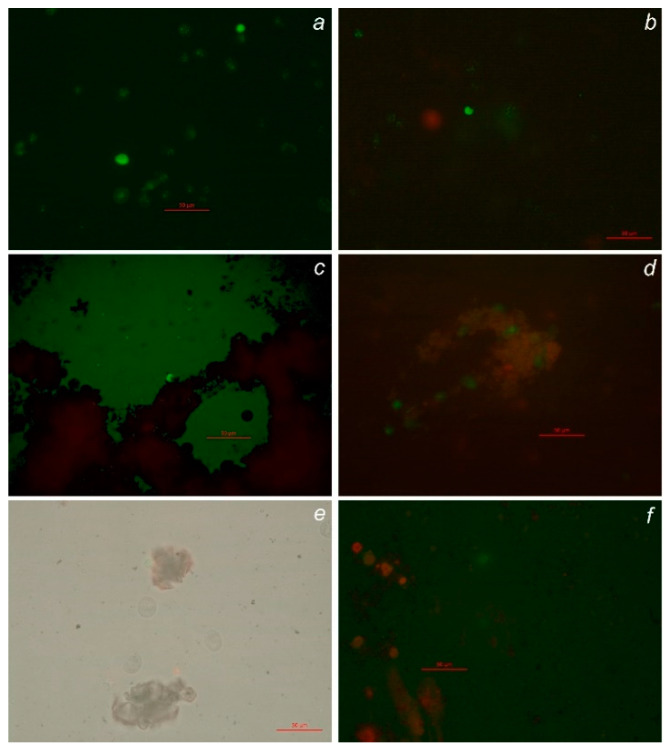
The hemocyte cells of the mollusc *A.boucardi* after 6 h of exposure with the NPs at the concentration of 1000 mg/L: (**a**) control; (**b**) CNT-2; (**c**) CdS; (**d**) ZnS; (**e**) Au-NPs; and (**f**) TiO_2_.

The observed changes in the hemocyte cells revealed a cytotoxic effect, according to the data in Figure 1, represented in Appendix B. The cells of *C. grayanus* exposed to 1000 mg/L of the samples CdS, ZnS, and TiO_2_ over 6 h are shown in Figure A1. The hemocytes of *M. modiolus* exposed to CNT-1, CNT-2, SNT-1, CdS, ZnS, Au-NPs, and TiO_2_ at the same conditions are presented in Figure A2. The changes of *A. boucardi* cells after 6 h of treatment with CNT-2, CdS, ZnS, Au-NPs, and TiO_2_ are shown in Figure A3.

High mortality and the residuals of dead *C. grayanus* hemocytes exposed to ZnS NPs are represented in Figure A1c. A similar observation was noticed for the cells of *M. modiolus* exposed to Au-NPs (Figure A2g) and for the cells of *A. boucardi* exposed to ZnS NPs (Figure A3d).

## 4. Discussion

The available literature confirmed the applicability of bivalve mollusc species as a reliable model for both in vivo and in vitro bioassays in nanotoxicology [29,30]. However, the species chosen in our study are not commonly used for NP toxicity evaluation [68,69]. In this study, we have identified the differences between the influence of 10 types of carbon, silicon, and metal-based NPs on hemocytes of three typical marine mussels of the Sea of Japan, namely, *C. grayanus*, *M. modiolus*, and *A. boucardi* [45]. 

### 4.1. Toxicity of Carbon Nanotubes and Nanofibers on Bivalve Molluscs

The absence of any visible cytotoxic influence of carbon nanofibers CNF-1 and CNF-2 on hemocytes of all the tested species (Figure 1) could be because of the lower bioavailability of the samples according to their surface and structural properties [70,71]. Moreover, the dose- and time-dependent increase in the mortality for *M. modiolus* exposed to carbon nanotubes CNT-1 and CNT-1 (Figure 1d–f), and the increase in the mortality for *A. boucardi* exposed to CNT-2 (Figure 1i), are apparently linked with the differences in the structure of used carbon NPs (Table 1). At the same time, there was no correlation between registered toxic effects and the composition of chemical impurities in the samples. A similar observation was previously reported for hemocytes of the mussel *M. edulis* exposed to carbon nanotubes and carbon nanofibers [72]. Furthermore, the higher toxic effect of single-walled carbon nanotubes in comparison with multi-walled carbon nanotubes was observed for the cells of *Mytilus* sp. [73], which confirms the higher toxicity of more hydrophilic carbon NPs. In our early work with the same samples of NPs tested on four microalgae species [74], we had shown that surface hydrophobicity of the sample and its affinity to a cell wall of marine organisms play a key role in the level of carbon NP aquatic toxicity. Therefore, it seems that more hydrophilic carbon nanotubes CNT-1 and CNT-1 generated higher mechanical damage to the hemocyte cells compared with hydrophobic and less ordered carbon nanofibers CNF-1 and CNF-2. In particular, the reliability of this proposition can be seen for the *M. modiolus* hemocytes (Figure 1d,e), as this species proved to be the most sensitive used test-object.

Moreover, the sensitivity of *M. modiolus* hemocytes exposed to the NPs was expressed in terms of the highest membrane depolarization as compared with the other two species (Figure 2). All the tested samples of carbon NPs affected membrane polarization of hemocytes of all used species in a dose-dependent manner. The reduction of the membrane potential was previously described as an indicator of pre-apoptotic processes and as a marker of early sub-lethal toxicity [75]. The tolerance of *C. grayanus* cells to the influence of the tested carbon NPs (Figure 1a–c) correlates with the lowest level of membrane depolarization as compared with the other two species (Figure 2). Besides, *A. boucardi* revealed a cytotoxic response after 6 h of exposure to CNT-2 NPs (Figure 1i) and demonstrated a higher membrane depolarization at the concentrations of 500 and 1000 mg/L (Figure 2).

These observations allow us to hypothesize that a longer exposure of hemocytes with carbon NPs could lead to further membrane depolarization and, finally, could result in cytotoxicity. This is an important foundation for prolonged exposure studies in the future.

### 4.2. Toxicity of Silicon Nanotubes on Bivalve Molluscs

Only the hemocytes of *M. modiolus* demonstrated a cytotoxic response under the influence of SNT-1 after 6 h of exposure (Figure 1f). The higher toxicity of SNT-1 as compared with SNT-2 NPs is correlated with a smaller size and a significantly more developed surface area (Table 1). Higher toxicity of smaller particles with a bigger surface area is one of the key points in particle toxicology [76].

The analysis of changes in hemocyte membrane polarization showed that SNT-1 NPs had a more significant effect on membrane charge of all three mussel species as compared with SNT-2 NPs (Figure 2). Interestingly, the hemocytes of *A. boucardi* responded with dose-dependent membrane hyperpolarization, which is possibly because of the early activation of several cell death pathways [77,78]. The most pronounced membrane depolarization was observed for the *M. modiolus* cells exposed to SNT-1 owing to their smaller size (Figure 2). These observations agree with the registered cytotoxic effect of the SNT-1 NPs (Figure 1f).

According to the cytotoxicity data (Figure 1f), we can emphasize the similarity between the shapes of viability-curves for hemocytes of *M. modiolus* exposed to carbon nanotubes (CNT-1 and CNT-2) and silicon nanotube sample (SNT-1). These findings suggest a similar mode of toxic action for carbon and silicon nanotubes, which is most probably expressed in the mechanical influence of the particles to the cells.

### 4.3. Toxicity of Metal-Based Nanoparticles on Bivalve Molluscs

#### 4.3.1. Metal Sulfide Nanocrystals

Metal sulfide nanocrystals, that is, CdS and ZnS, were previously described as highly toxic NPs for the aquatic organisms [79,80,81] because of their small size, photoactivity under visible and UV-light, and possible release of toxic metal ions as a result of nanocrystal photocorrosion in water [82,83]. We registered dose- and time-dependent cytotoxicity of CdS and ZnS NPs to hemocytes of all three bivalve species (Figure 1). A significant increase of ZnS NP toxicity could be observed at 6 h measurements for *C. grayanus* (Figure 1c) and *A. boucardi* (Figure 1i). The observed effect is possibly linked with the lower stability of ZnS nanocrystals in water (log K_s0_, –26.02) as compared with that of CdS (log K_s0_, –31.42), which results in nanocrystal solubility (e.g., more Zn^2+^ and S^2-^ ions can be formed in solution) [83]. The higher aquatic toxicity of Zn^2+^ ions as compared with the NP form was described in previous works [55,84]. Moreover, the superior toxicity of CdS and ZnS NPs might be the result of a relatively smaller size as compared with the other NPs (Table 1).

Significant membrane depolarization of hemocytes exposed to CdS and ZnS NPs (Figure 2) is probably caused by a high photoactivity of metal sulfide nanocrystals [60], which facilitates the generation of reactive oxygen species (ROS) in the media and, finally, leads to the oxidative damage and general cellular metabolic disorders [85,86]. Similarly, it was previously described that Zn^2+^ ions lead to intracellular ROS generation and cause mitochondrial membrane depolarization and dysfunction [87].

Despite the high level of toxicity registered for CdS and ZnS NPs, there are still many unanswered questions about the correlation between the aquatic toxicity of NPs and parameters such as light irradiation, ROS formation, and transformation of the NPs. Further research is needed to examine more closely the links between particle characteristics and their toxic influence on aquatic organisms.

#### 4.3.2. Titanium Dioxide Nanoparticles

TiO_2_ NPs are used in a variety of consumer products and are widely applied in environmental purification [88,89]. Previous studies have demonstrated that the anatase crystal form of TiO_2_ NPs is significantly more toxic than the rutile particles [90]. Contrary to *C. grayanus* and *A. boucardi* cells, the *M. modiolus* hemocytes responded with membrane hyperpolarization under the influence of TiO_2_ NPs (Figure 2). Such a response could be because of the ROS generation by photoactive TiO_2_ NPs [91] and further oxidative disorder. The differences observed in the toxic effects of these NPs between the tested species could be associated with the dissimilarity in their antioxidative defense systems, as previously shown for several bivalves [92]. The *A. boucardi* hemocytes responded to TiO_2_ NPs only after 6 h of exposure at the highest concentrations (Figure 1i). Therefore, it can be assumed that the longer exposure of TiO_2_ NPs could cause a significant increase in a toxic effect on the hemocytes of tested mollusc species. A further study should assess the long-term influence of TiO_2_ NPs on marine bivalves.

#### 4.3.3. Gold Nanoparticles

Gold NPs have promising applications in medicine, biology, and chemistry [93]. Previous work did not show short-term and sub-chronic toxicity of citrate-stabilized Au-NPs on marine bivalve *R. philippinarum* [94]. Another study demonstrated the accumulation of Au-NPs in *M. edulis* bivalve mollusc and oxidative stress-related toxicity [95]. Our results have demonstrated the cytotoxic influence of Au-NPs on hemocytes of *M. modiolus* (Figure 1d–f) and *A. boucardi* (Figure 1g–h). Interestingly, the hemocytes of *C. grayanus* revealed tolerance to Au-NPs after short-term exposure (Figure 1a–c), but showed significant membrane depolarization (Figure 2). 

## 5. Conclusions

The highest aquatic toxicity was registered for metal-based NPs, which caused cytotoxicity to the hemocytes of all the studied bivalve species. Our results demonstrated that more hydrophilic carbon nanotubes CNT-1 and CNT-1 generated higher mechanical damage to bivalve hemocyte cells compared with hydrophobic and less ordered carbon nanofibers CNF-1 and CNF-2. The higher toxicity of SNT-1 as compared with SNT-2 NPs is correlated with a smaller size and a significantly more developed surface area of the sample. Moreover, the similarity between the shapes of viability-curves for the hemocytes exposed to carbon nanotubes and silicon nanotubes suggest a similar mode of toxic action for carbon and silicon nanotubes, which is most probably expressed in the mechanical influence of the particles to the cells. We believe that the results of this short-term in vitro bioassay will lay the foundations for further understanding of the risks associated with NPs and provide guidance for choosing bivalve molluscs as the tested species for such risk assessment studies in the area of aquatic nanotoxicology. This finding, while preliminary, represents an interesting case for further research. We intend to extend this study by applying indicators of oxidative stress and the implementation of long-term exposure.

## Figures and Tables

**Table 1:** Characteristics of the nanoparticles used in the experiment. CNT, carbon nanotube; CNF, carbon nanofibers; SNT, silicon nanotube; NP, nanoparticle.

Sample	Diameter nm	Surface Area m^2^/g	Impurities %	Structural Features
CNT-1	18–20	130	Al–0.9	many particles with defect areas and opened ends of carbon nanotubes
			Co–0.3	
			Fe–0.6	
CNT-2	18–20	150	Ca–0.004	ordered nanotube structure
			Cl–0.08	
			Co–0.12	
			Fe–0.2	
CNF-1	90–120	90–100	Al_2_O_3_–0.4	unordered structure, defect areas, the presence of amorphous carbon
CNF-2	90–120	90–100	Al_2_O_3_–0.4	unordered structure, defect areas
			Ni–3.6	
SNT-1	3–4	685	—	ordered nanotube structure
SNT-2	40–45	395	—	ordered nanotube structure
CdS	5–9	n/a	—	cubic crystal phase
ZnS	2.6–5.6	n/a	—	cubic crystal phase
Au-NPs	60-80	n/a	—	spherical shape
TiO_2_	32	45	total metal–0.1	anatase crystal structure

## Appendix A

**Table A1:** Calculated values of EC_50_ for viability of the hemocytes after the exposure to the nanoparticles, mg/L.

NPs	*C. grayanus* ^1^			*M. modiolus* ^2^			*A. boucardi* ^3^		
	2 h	4 h	6 h	2 h	4 h	6 h	2 h	4 h	6 h
CNT-1	n/a	n/a	n/a	864.6	512.2 (366.1–670.9)	241.5 (132.9–382.5)	n/a	n/a	n/a
CNT-2	n/a	n/a	n/a	471.1	447.3 (319.5–672.6)	284.7 (136.7–459.9)	n/a	n/a	752.6
CNF-1	n/a	n/a	n/a	n/a	n/a	n/a	n/a	n/a	n/a
CNF-2	n/a	n/a	n/a	n/a	n/a	n/a	n/a	n/a	n/a
SNT-1	n/a	n/a	n/a	n/a	n/a	606.8 (387.3–853.8)	n/a	n/a	n/a
SNT-2	n/a	n/a	n/a	n/a	n/a	n/a	n/a	n/a	n/a
CdS	616.7	620.8	637.9	827.3	530.3 (388.4–837.6)	212.4 (136.3–300.0)	752.6 (706.8–800.8)	662.9 (507.7–1233)	538.2 (383.9–824.2)
ZnS	275.5 (157.0–409.2)	277.4 (90.4–552.2)	143.4 (58.0–266.9)	268.6 (232.3–305.8)	283.5 (218.9–349.0)	251.2 (178.1–321.7)	n/a	157.8 (83.6–270.4)	147.9 (123.8–172.0)
Au-NPs	n/a	n/a	n/a	n/a	591.8	560.5 (528.3–591.9)	n/a	766.8	597.1 (482.4–758.7)
TiO_2_	343.6 (234.3–518.0)	368.1 (306.9–433.9)	338.8 (210.5–476.5)	333.6 (253.0–448.5)	354.0 (317.6–394.7)	376.6 (297.4–455.1)	n/a	n/a	n/a

95% confidence limits presented in the parentheses. n/a—measured effect was not observed even at the highest concentrations of the sample (1000 mg/L). ^1^ *p* = 0.0078. ^2^ *p* = 0.0267. ^3^ *p* < 0.0001.
